# Slow-release boron fertilizer improves yield and nutritional profile of *Beta vulgaris* L. grown in Northeast China by increasing boron supply capacity

**DOI:** 10.3389/fpls.2024.1441226

**Published:** 2024-12-16

**Authors:** Zhenzhen Wu, Xiaoyu Zhao, Jean Wan Hong Yong, Shafaque Sehar, Muhammad Faheem Adil, Muhammad Riaz, Krishan K. Verma, Meiyu Li, Jialu Huo, Songlin Yang, Baiquan Song

**Affiliations:** ^1^ National Sugar Crops Improvement Center, Ministry of Education & Heilongjiang Provincial Key Laboratory of Ecological Restoration and Resource Utilization for Cold Region, Heilongjiang University, Harbin, China; ^2^ Sugar Beet Engineering Research Center Heilongjiang Province, Ministry of Education & Heilongjiang Provincial Key Laboratory of Ecological Restoration and Resource Utilization for Cold Region, Heilongjiang University, Harbin, China; ^3^ Engineering Research Center of Agricultural Microbiology Technology, Ministry of Education & Heilongjiang Provincial Key Laboratory of Ecological Restoration and Resource Utilization for Cold Region, Heilongjiang University, Harbin, China; ^4^ Department of Biosystems and Technology, Swedish University of Agricultural Sciences, Alnarp, Sweden; ^5^ College of Agriculture and Biotechnology, Zhejiang University, Hangzhou, China; ^6^ College of Resources and Environment, Zhongkai University of Agriculture and Engineering, Guangzhou, China; ^7^ Key Laboratory of Sugarcane Biotechnology and Genetic Improvement (Guangxi), Guangxi Academy of Agricultural Sciences, Nanning, Guangxi, China; ^8^ Ministry of Agriculture and Rural Affairs/Guangxi Key Laboratory of Sugarcane Genetic Improvement/Sugarcane Research Institute, Guangxi Academy of Agricultural Sciences, Nanning, Guangxi, China

**Keywords:** yield, quality, novel fertilizer, sugar beet, black soil, albic soil

## Abstract

The northeastern part of China is a traditional sugar beet cultivation area where the soils are classified generally as the black and albic soil types with low boron (B) availability. Boron fertilizer can increase soil B content and significantly improve crop yield and quality. At present, the effects of slow-release B fertilizer on beet root yield and quality remain unclear. Two sugar beet varieties KWS1197 and KWS0143 were selected as the research materials; and biologically evaluated with three dosage rates of 0, 15, and 30 kg ha^-1^ in two soil types. Results showed that slow-release B fertilizer (30 kg ha^-1^) improved sugar beet net photosynthetic rate (13.6%) and transpiration rate (9.8%), as well as enhanced dry matter accumulation and the transfer to underground parts (23.1%) for higher root yield (1.4 to 9.7% in black soil and 3.5-14.2% in albic soil). Specifically, boron fertilizer greatly increased root B accumulation, as evidenced by decreasing amino N and Na contents alongside increasing surose (Pol) content. Slow-release B fertilizer increased white sugar yield by 3.5 to 35.7% in black soil and 5.8 to 20.8% in albic soil. In conclusion, applying slow-release B fertilizer is an effective strategy to increase sugar beet yield and quality in northeast China, with a recommended application rate of 30 kg ha^-1^. These findings established a baseline for formulating effective and futristic fertilizer for sugar beet.

## 1 Introduction

Boron (B) is an essential trace element for crop growth, playing crucial roles in photosynthesis, carbohydrate metabolism, and sugar transport (Fujiyama et al., 2019; Kohli et al., 2023). Numerous studies have found that B deficiency reduces crop photosynthetic performance and dry matter accumulation, resulting in decreased crop yield (Li et al., 2017; Chen et al., 2023). The shortage of available soil B is a global issue (Wang et al., 2015), affecting regions across Asia, Oceania, Africa, America, and Europe (Lehto et al., 2010), with 132 plant species in 80 countries exhibiting symptoms of B deficiency (García-Sánchez et al., 2020). According to statistics from China’s second national soil survey, more than 3×10^7^ ha of China’s arable land is classified as B deficient soil (<0.5 mg kg^-1^) (Wei et al., 2022). Boron fertilizer has now become the largest trace element fertilizer used in agricultural production in China.

Sugar is essential for the nutrition of human body, providing approximately 70% of the energy needed to maintain physiological functions (Chung et al., 2022). Sugar beet contributes about 30% of global sugar production, with China accounting for 10% of its domestic sugar production (Wang et al., 2019). Sugar beets are mostly grown in North China, Northwest China and Northeast China (Xie et al., 2022). Northeast China is one of the world’s four main black soil belts, with significant temperature variations from day to night, abundant rainfall, and fertile soil, which make it a traditional beet production region (Hou and Wang, 2022). Sugar beet cultivation began in Northeast China in 1905, with a maximum planting area of 330,000 ha (Du et al., 2022). In recent years, due to the increase in crop yield levels and insufficient attention to micronutrient input, soil fertility in this area has declined, especially in B content, which is inadequate to meet sugar beet production demands (Zhu et al., 2020). The average boron content of beet soil in Northeast China is reported to be 0.39 mg kg^-1^, and B deficiency is the main limiting factor restricting beet productivity in this region (Song et al., 2022).

Sugar beet is one of the most sensitive crops to B availability and has a strong requirement for this micronutrient (Cooke and Scott, 2012). In field production, B deficiency generally lowers sugar beet growth; in severe cases of poor availability, the phenomenon of “heart rot” is prevalent and can lead to crop failure (Gordon et al., 2022). Compared with traditional borax and other fertilizers, slow-release B fertilizers have the advantages of high efficiency and environmental friendliness, which can better satisfy the demand for B nutrient during the middle and later growing stages, thus better promoting crop growth and improving B utilization, as well as effectively reducing the fertilizer pollution in the environment (Zheng et al., 2023). At the moment, the application of slow-release B fertilizers to crops, like rapeseed (Abat et al., 2015) and corn (Duboc et al., 2021), has shown notable positive impacts on yield and quality. Nevertheless, it is uncertain how slow-release B fertilizer affects beet root quality in albic and black soils.

Therefore, a field trial was conducted with two sugar beet varieties KWS1197 and KWS0143 in the black soil zone of Northeast China. The goals were: (i) to investigate the impacts of slow-release B fertilizer on photosynthesis, dry matter accumulation, and boron translocation efficiency in beet, (ii) to evaluate the effect of slow-release B fertilizer on root yield improvement, and (iii) to elucidate the mechanism by which slow-release B fertilizer affects root quality and white sugar yield. This research provides a theoretical framework for increasing beet sugar yield and applying novel fertilizers.

## 2 Materials and methods

### 2.1 Experimental sites

**Figure 1:**
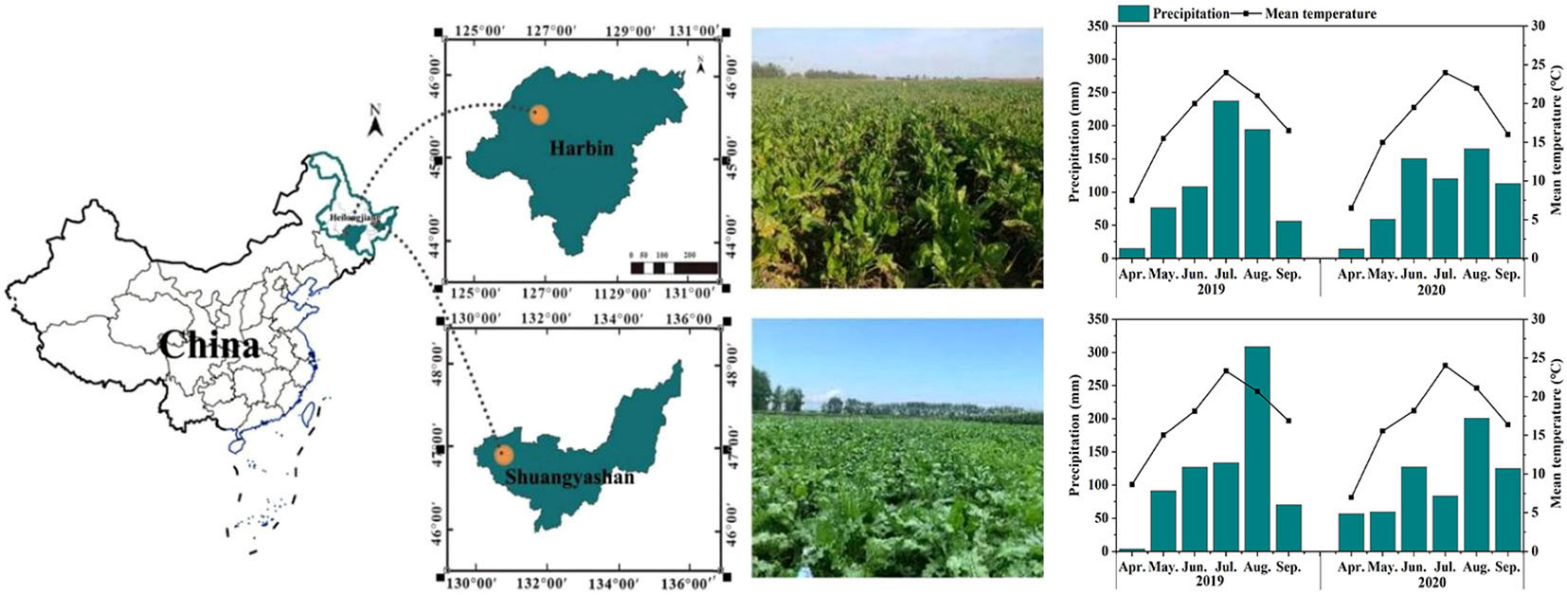
The experimental locations.

The experiment was conducted in the fields of the National Sugar Improvement Center, Hulan, Heilongjiang Province, China (46°08′N, 126°77′E), and Heilongjiang Academy of Agricultural Reclamation Sciences, Baoshan, Heilongjiang Province, China (45°35′N, 129°55′E), during 2019-2020 (**Figure 1**). Both regions have a temperate continental monsoon climate, with rain and heat occurring in the same season. According to China’s second soil census, the soil types in Hulan and Baoshan are black soil (BS) and albic soil (AS), and their soil chemical characteristics are listed in **Supplementary Table S1**.

### 2.2 Experimental treatments

**Figure 2:**
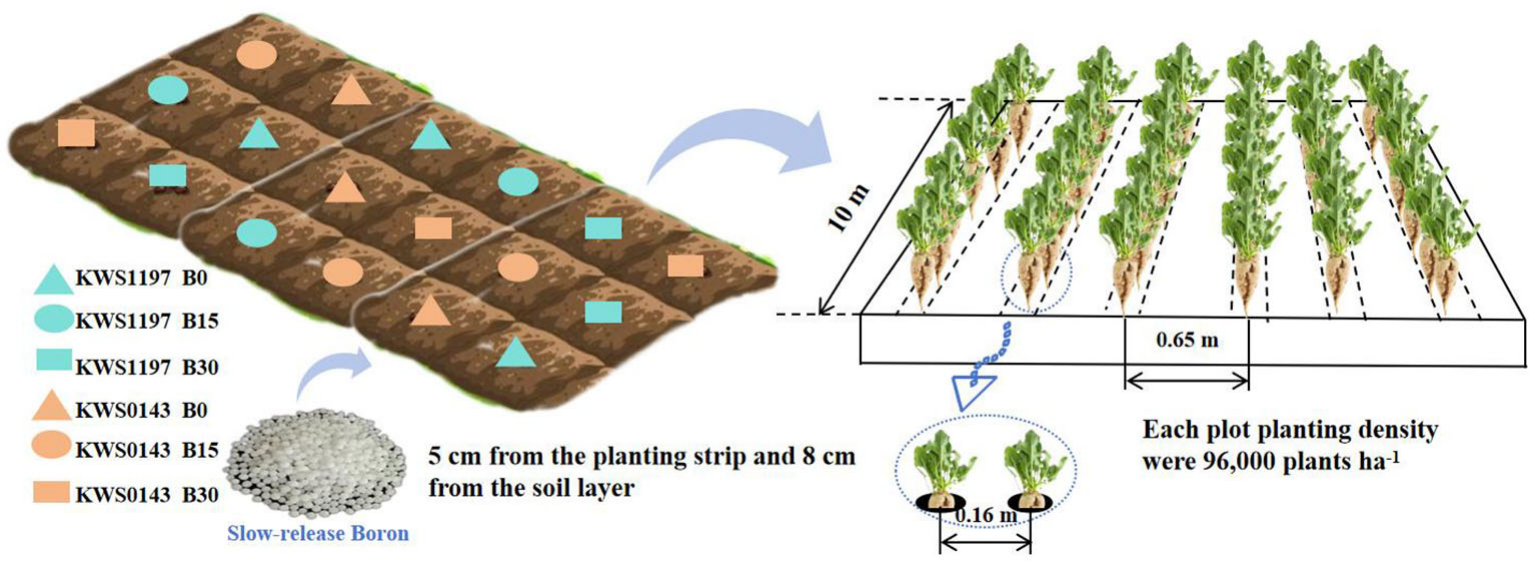
Schematic diagram of the cropping systems used in this study in China.

Sugar beet varieties (KWS1197 and KWS0143) were selected for the experiment, and three boron levels, i.e., B0 (0 kg ha^-1^, CK), B15 (15 kg ha^-1^), and B30 (30 kg ha^-1^) were devised. Each plot had six rows and was 10 m in length, covering about 40 m^2^. The plant spacing × row spacing was set at 16 cm × 65 cm, with a planting density of 96,000 plants ha^-1^. Urea (N 46%), diammonium phosphate (N 18%, P_2_O_5_ 46%), and potassium sulfate (K_2_O 50%) fertilizers were applied in each plot @ 110, 90, and 110 kg ha^-1^, respectively. The slow-release B fertilizer was granular, with a B content of 15% and a release period of 120 days (produced by Shandong Jinrunzi Biotechnology Co., Ltd). These fertilizers were combined and applied 5 cm from the planting strip and 8 cm deep in the soil layer (**Figure 2**).

### 2.3 Data collection

#### 2.3.1 Plant performance measurements

Ten fully extended mature leaves of similar length and orientation were chosen for data analysis. The net photosynthetic rate (*Pn*), stomatal conductance (*Gs*), transpiration rate (*Tr*), and intercellular CO_2_ concentration (*Ci*) were measured by a portable and open gas exchange photosynthetic system (CI-340, CID Bioscience, USA) on September 26, 2020 (Yong et al., 2010, 2014); the gas exchange calculations were in accordance to Farquhar et al. (1980). The upper leaves were selected for measuring the respective parameters between 10:00 a.m. and 12:00 p.m (Yong et al., 2014). Relative chlorophyll content (SPAD) was assessed utilizing a SPAD-502 meter (Yong et al., 2010). The leaf area was recorded by a scanner, and calculated using imageJ analysis software, then leaf area index (LAI) was calculated (**Supplementary Table S2**) (Wu et al., 2021; Zhao et al., 2023).

#### 2.3.2 Biomass measurement

Sugar beet was sown in the last week of April. Sampling dates were June 20, July21, August 24, and September 21 in 2019, and September 26 in 2020. Samples (200 g) of various plant parts, such as green leaves and petioles, roots, senescent leaves, and petioles, were dried in the oven at 60 ± 2°C.

#### 2.3.3 Analysis of total boron content

The dry sample was weighed (200 mg) and placed in a quartz crucible, then ashed at 560°C for 4 hours, followed by an extraction with 0.1 M hydrochloric acid (HCl) for 0.5 hour. The B content was evaluated through dry ashing-curcumin colorimetry (Dible et al., 1954). A double-beam UV-spectrophotometer (UV-8000 A, Shanghai, China) was used to determine B content, and B accumulation and its transfer coefficient were calculated according to the following formula:


$$B\;accumulation\;\left(g\;ha^{-1}\right)\;=1000\;\times \;biomass\;of\;each\;part\;\left(kg\;ha^{-1}\right)\;\times \;B\;content\;of\;each\;part\;\left(mg\;kg^{-1}\right)$$

(Song et al., 2019).


$$Boron\;transfer\;coefficient\;\left(BTC\right)\;=\;aboveground\;accumulation\;(g\;ha^{-1})\;/\;belowground\;accumulation\;\;(g\;ha^{-1})$$

(Wang et al., 2023).

#### 2.3.4 Root yield and quality

At the end of September 2019 in both and 2020, the root yield and quality of the experimental fields were measured. The number and weight of roots were assessed according to the harvested area along with the number of plants and yield. At the same time, ten representative roots were taken from each plot and brought back to the laboratory for washing. Samples of 20 g from each root were prepared into a paste, which was used to identify root quality, such as sucrose (Pol), amino N, Na, K, and white sugar content (WSC); moreover, white sugar yield (WSY) was also estimated (**Supplementary Table S2**) (Götze et al., 2017).

### 2.4 Analytical method

SPSS version 28 was used to analyze the data with a one-way analysis of variance (ANOVA). When the data exhibited a normal distribution and homogeneity of variance, the general linear model was employed, with B fertilizer and soil type as fixed components and year as a random variable. When the data did not conform to a normal distribution or showed heterogeneous variance, the generalized linear model was employed for evaluation. Pearson's correlation test was applied to assess relationships, displaying only strong correlations. In due course, Origin 2021 was utilized for drawing diagrams.

## 3 Results

### 3.1 Boron levels influenced plant performance at the leaf level

**Figure 3:**
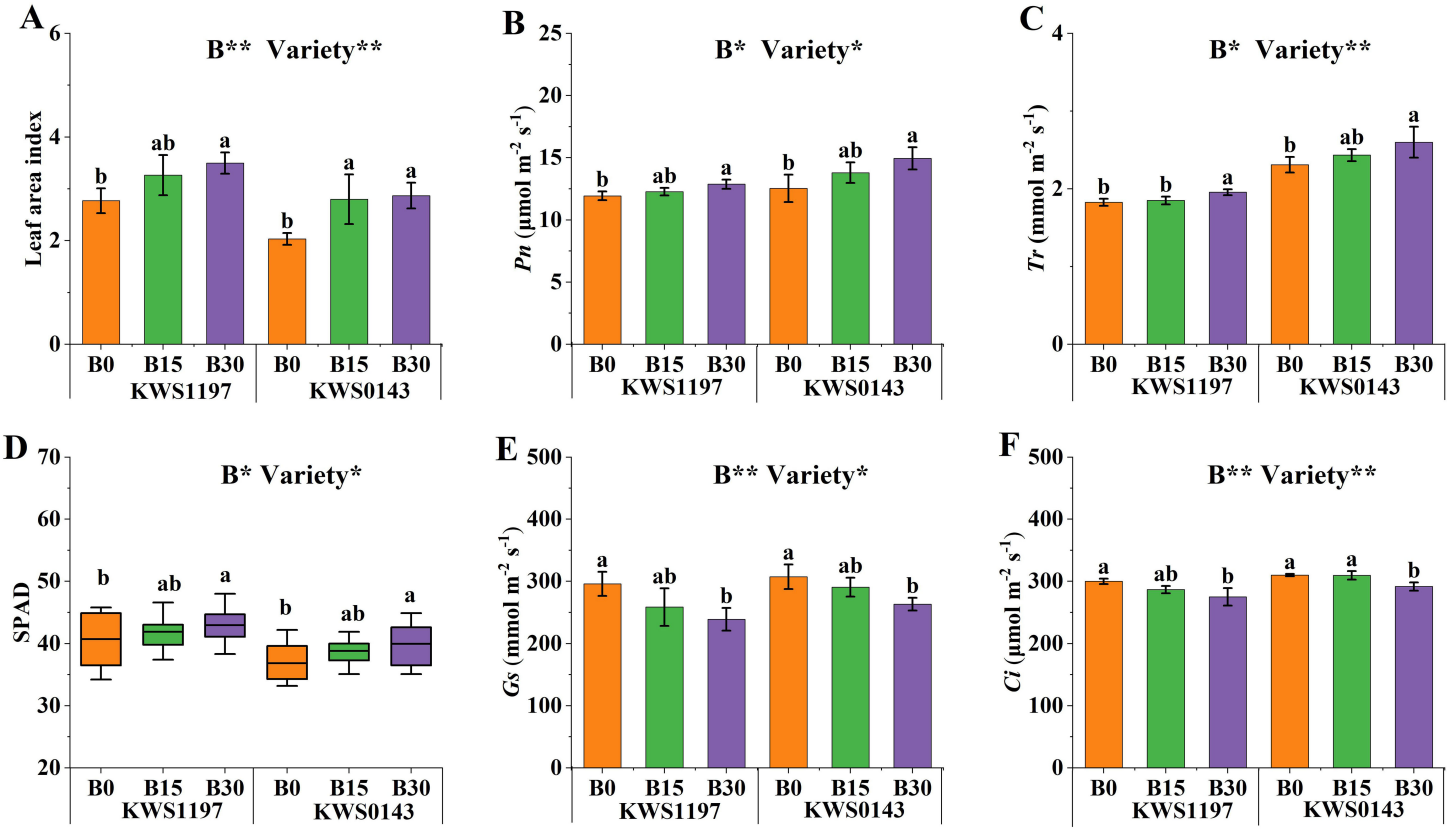
Effects of slow-release B fertilizer on leaf area index (LAI; **A**), net photosynthetic rates (Pn; **B**), transpiration rates (Tr; **C**), foliar chlorophyll content (SPAD; **D**), stomatal conductance (Gs; **E**), and intercellular CO_2_ concentration (Ci; **F**) of beet in the black soil in 2020. Values with various lowercase letters indicate significant variations.

The results showed that slow-release B fertilizer increased the leaf area index (LAI), net photosynthetic rate (*Pn*), transpiration rate (*Tr*), and relative chlorophyll content (SPAD) in both varieties reaching a maximum value (observed across treatments) with B30 treatment (**Figures 3A–D**). Compared with B0, SPAD, *Tr*, *Pn*, and LAI increased significantly, i.e., up to 7.0%, 9.8%, 13.6%, and 33.7% on average, respectively, under the B30 treatment. Patterns of intercellular CO_2_ concentration (*Ci*) and stomatal conductance (*Gs*) were consistent, with notable reductions upon with B application (**Figures 3E, F**). Furthermore, *Ci* and *Gs* reduced significantly (by 7.1% and 16.8%, respectively) under B30 treatment compared to B0. Under the same level of B application, the LAI and SPAD of the KWS1197 were higher, whereas *Pn*, *Tr*, *Ci*, and *Gs* were lower than that of KWS0143.

### 3.2 Genotypic differences and B availability affect dry matter accumulation, and boron transport efficiency across different soil types

**Figure 4:**
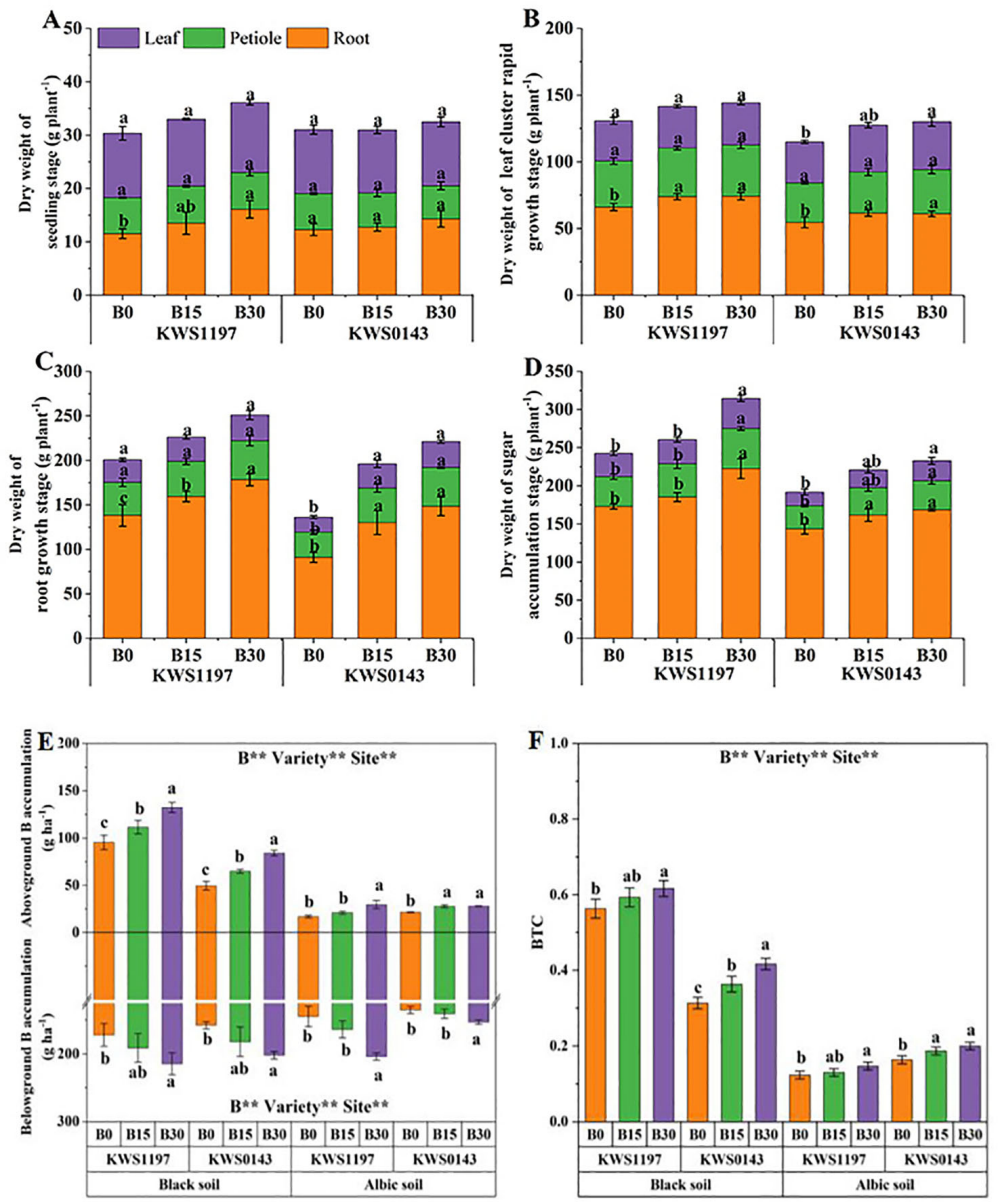
Effects of slow-release B fertilizer on sugar beet dry matter distribution during the seedling stage **(A)**, leaf cluster rapid growth stage **(B)**, root growth stage **(C)**, and sugar accumulation stage **(D)** in black soil in 2020; as well as levels of boron **(B)** accumulation **(E)**, and BTC **(F)** in sugar beet growing in different soil categories during the 2020 harvest period. Values with various lowercase letters show significant variations.

Slow-release B increased the dry weight of roots, petioles, and leaves during the sugar accumulation period, reaching a maximum under B30 treatment, with significant increases of 23.1%, 29.6%, and 37.5%, respectively. Under the same B application, KWS1197 variety accumulated more dry matter than KWS0143 (**Figures 4A–D**).

The B accumulation in both beet varieties across the two soil types increased significantly with B application. This enhancement was more pronounced in the root part, with a significant rise of 24.8% and 28.1% in the black soil, as well as 41.0% and 13.2% in the albic soil for KWS1197 and KWS0143, respectively (**Figure 4E**). Furthermore, after B application, the boron transfer coefficient (BTC) of the B30 treatment showed a substantial increase of 9.5% and 33.0% in the black soil and 18.9% and 22.5% increase in the albic soil for KWS1197 and KWS0143, respectively (**Figure 4F**).

### 3.3 Boron improved root yield and quality

The interaction among soil type, variety, and slow-release B fertilizer exhibited a substantial impact on white sugar yield (WSY) and root yield (RY). Slow-release B notably increased the RY and BSY of both sugar beet varieties in both soil types compared to plants without B application. In the black soil, the RY and WSY of KWS1197 increased by 2.9-9.7%, 7.1-30.6%, respectively, whereas for KWS0143, a respective increase of 1.4-9.6% and 3.5-35.7% was observed. Similarly, in the albic soil, the RY and WSY of KWS1197 increased by 3.9-9.5% and 5.8-20.8%, meanwhile for KWS0143, an increase of 3.5-14.2% and 8.5-20.1% was exhibited, respectively (**Table 1**).

**Table 1:** Effects of slow-release B fertilizer on beet root K, amino N, Na, and sucrose (Pol) content, white sugar content (WSC), root yield (RY), and white sugar yield (WSY) in two soil type at the harvesting stage.

Soil types	Variety	Treatments	K		Na		amino N		Pol		WSC		RY		WSY	
			mmol (100 g)^-1^						(%)				(t ha^-1^)			
			2019	2020	2019	2020	2019	2020	2019	2020	2019	2020	2019	2020	2019	2020
Black soil	KWS1197	B0	3.73b	6.51c	3.25a	4.38a	2.26a	8.05a	13.51c	9.76b	11.05b	5.44b	44.84b	52.89b	4.96b	2.88c
		B15	3.54c	7.32b	3.25a	3.01b	1.33b	7.72a	13.72b	10.29ab	11.51ab	6.11a	46.17ab	54.42b	5.31ab	3.32b
		B30	4.06a	7.83a	2.85b	2.91b	1.90ab	7.81a	13.99a	10.72a	11.62a	6.48a	47.70a	58.04a	5.54a	3.76a
		Mean	3.78	7.22	3.12	3.43	1.83	7.86	13.74	10.25	11.39	6.01	46.23	55.12	5.27	3.32
	KWS0143	B0	2.76b	8.48a	5.92b	4.51a	1.40a	9.78a	10.67b	8.78b	8.21b	3.79c	38.57c	51.59b	3.19b	1.96b
		B15	3.28a	8.24a	5.86b	4.13a	1.16b	7.95b	10.75b	9.05b	8.29b	4.58b	40.08b	52.29ab	3.30b	2.40b
		B30	2.74b	8.45a	5.39a	4.22a	1.32ab	6.99b	11.52a	9.37a	9.15a	5.03a	42.27a	52.91a	3.86a	2.66a
		Mean	2.93	8.39	5.72	4.29	1.29	8.24	10.98	9.37	8.55	4.79	40.31	52.26	3.45	2.51
Albic soil	KWS1197	B0	4.60a	3.35b	1.46a	0.55a	1.69a	1.69a	16.32b	16.80b	14.10b	14.85b	26.18b	44.04b	3.69c	6.54c
		B15	3.81b	3.83a	1.31a	0.53a	1.64a	1.66ab	17.09b	17.12b	15.00a	15.12b	27.60ab	45.74a	4.14b	6.92b
		B30	4.54a	3.94a	1.36a	0.40b	1.32b	1.39b	17.35a	18.91a	15.24a	16.97a	28.67a	46.52a	4.37a	7.90a
		Mean	4.31	3.71	1.38	0.49	1.55	1.58	16.92	17.61	14.78	15.65	27.48	45.44	4.07	7.12
	KWS0143	B0	4.84a	3.27b	2.69a	1.33a	2.56a	1.68a	15.41b	16.28b	12.81c	14.24b	25.82b	40.50 b	3.31b	5.77b
		B15	4.44b	3.56a	2.52a	1.04b	2.10ab	1.65ab	15.85b	16.47b	13.43b	14.45b	26.71ab	44.40ab	3.59ab	6.41ab
		B30	3.94c	3.72a	2.05b	0.95b	1.52b	1.50b	16.58a	17.00a	14.42a	15.00a	27.28a	46.23a	3.93a	6.93a
		Mean	4.41	3.50	2.42	1.11	2.06	1.61	15.95	16.58	13.55	14.56	26.60	43.71	3.61	6.37
Source of variation *P* value																
Soil categorie (S)			**		**		**		**		**		**		**	
Variety (V)			ns		**		ns		**		**		**		**	
Year (Y)			**		**		**		**		**		**		**	
Boron treatment (B)			**		**		**		**		**		**		**	
S × V× B× Y			**		**		**		**		**		**		**	

Values with different lowercase letters are significantly different in the same soil type according to the least significant difference test. * *P*< 0.05, ** *P*< 0.01; ns, non-significant.

Slow-release B fertilizer decreased the amino N and Na contents in root grown in two soil categories, whereas the white sugar content (WSC) and sucrose (Pol) rose in comparison to B0. Moreover, the Pol and WSC of sugar beet planted in albic soil were significantly greater than that in black soil, contrary to the amino N and Na contents, which were notably decreased in albic soil compared to black soil for two consecutive years (**Table 1**).

### 3.4 Correlation analysis

**Figure 5:**
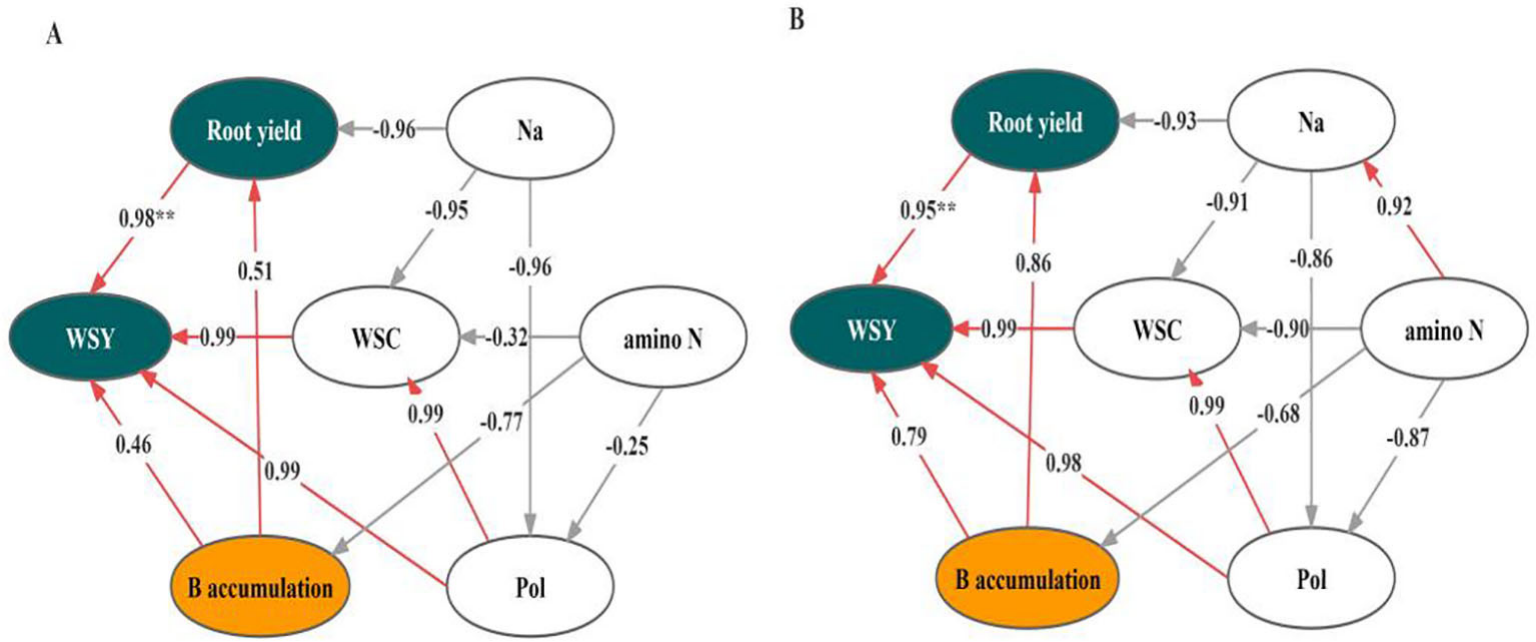
Correlation analysis between white sugar yield (WSY) and root chemical quality in black soil (BS; **A**) and albic soil (AS; **B**). WSC represents white sugar content, and Pol represents sucrose. The figures beside the arrows denote correlation coefficients. Red arrows indicate positive relationships, while gray arrows indicate negative relationships.

The correlation analysis of both soil categories revealed that WSY was positively related to root yield, WSC, and root B accumulation. Additionally, WSC was found to be negatively connected with K content in the black soil, alongside amino N and Na contents in the albic soil. This demonstrated that novel fertilizer increased the WSC by decreasing root ash content, resulting in a rise in WSY, but the ash content changed differently in both types of soils (**Figure 5**).

## 4 Discussion

Current research found that the basal application of granular slow-release B fertilizer increased the sugar beet leaf area index, which proved conducive to maintaining photosynthetic assimilation capacity during the middle and later growing stages, providing a physiological basis for increased dry matter. Specifically, boron fertilizer increased relative chlorophyll content (SPAD), and enhanced the photosynthetic activity of mesophyll cells, resulting in an improvement in gas exchange characteristics (net photosynthetic and transpiration rates) and, eventually, in CO_2_ assimilation capacity. Previous studies on rice (Calderón-Páez et al., 2021) and soybean (Galeriani et al., 2022) have also documented a similar response, where the application of B fertilizer markedly enhanced photosynthetic rates. In alignment with prior findings, our study further confirms that the new granular slow-release B fertilizer can improve the light assimilation ability of sugar beet, which is harvested for its underground biological yield.

High crop yield requires not only a large accumulation of dry matter, but also an efficient transport and distribution of dry matter among organs, which are crucial to the formation of economic yields (Zhang et al., 2023). For instance, investigations involving potato (Sarkar et al., 2018), radish (Maurya et al., 2019), and peanut (Ghazimahalleh et al., 2022) have shown that B application considerably improved the root-shoot ratio, and promote the biomass accumulation in belowground parts, thereby increasing economic yield. The current findings further confirm that granular slow-B fertilizer increased total biomass in sugar beet and directed more biomass to the roots to achieve higher economic yields.

Sugar beet root yield is closely related to factors, such as soil available B content and soil type (Hanse et al., 2011; Mekdad and Shaaban, 2020). Previous studies have found that sugar beet grown on B-deficient soils (< 0.5 mg kg^-1^) can experience a yield decline, whereas the application of B fertilizer significantly increases beet root yield (Nemeata Alla, 2017; Kandil et al., 2020). However, common B fertilizers, such as borax, prone to leaching, which often leads to a lack of B supply and a reduction in fertilizer utilization rates during the middle and later growth periods. This limitation reduces production potential and can even trigger physiological disorders such as heart rot (da Silva et al., 2018). Compared to traditional B fertilizers, slow-release B fertilizers reduce B loss, their slow and continuous release of nutrients better matches the crop’s fertilizer demand characteristics, ensuring consistent B supply, and promoting yield improvement (Zheng et al., 2023). Slow-release B fertilizer has shown clear advantages in crops like potato (Sharma et al., 2019) and oilseed rape (Everaert et al., 2023). Our research further confirmed that applying an optimal amount of slow-release B fertilizer (30 kg ha^-1^) significantly improved beet yield in black soil (0.31 mg kg^-1^) and albic soil (0.37 mg kg^-1^), both of which have insufficient available B content. This improvement is attributed to the ability of slow-release granular B fertilizer to ensure a steady supply of B during the middle and late stages, thereby enhancing boron transport efficiency, increasing root B content, and ultimately boosting root yield.

In addition, soil properties such as clay minerals can also affect soil available B content, thereby limiting crop yields (Niaz et al., 2007; Tlili et al., 2019). It has been reported that available B in stagnic fluvisol (clay) soil environments with low organic matter, is easily adsorbed or forms complexes that restrict movement, resulting in B deficiency symptoms in beet, as evidenced by dry/brittle or wilted leaves, and lateral splitting of petioles, which severely suppresses root yield (Dridi et al., 2018). This study ascertained that slow-release B fertilizer could increase beet root yield in both soil types. However due to the clay-heavy texture of albic soil, which limits the effectiveness of B fertilizer, the application of slow-release B fertilizer is more beneficial in black soil. Nevertheless, due to variations in rainfall, accumulated temperature, soil B content, and the growth period of sugar beets in different regions, the scope of our research on B fertilizer has been limited to Northeast China. Therefore, we plan to extend future research to other regions of China.

**Figure 6:**
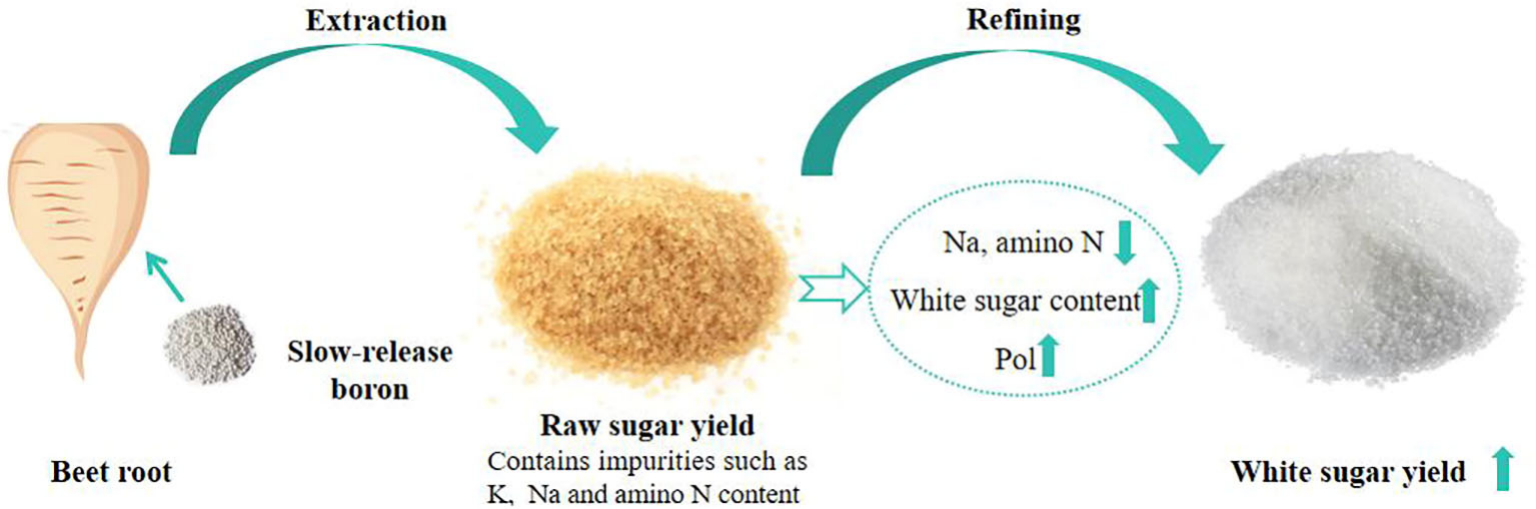
Schematic diagram illustrating the effects of slow-release B fertilizer on beet root chemical quality.

The primary objective of beet growth and production is to achieve the highest white sugar yield (Tian et al., 2023). The increase in white sugar yield is determined not only by beet yield and sugar content but also influenced by root ash content (K, Na, and amino N) (Artyszak et al., 2021). Therefore, these three factors in beet root are identified as key indicators of quality in beet processing, with elevated levels directly correlating with a decrease in sugar content (Abd El-Mageed et al., 2021). As early as 1935, boron application was discovered to improve beet root morphology and increase yield (Hughes and Murphy, 1935). Later studies further confirmed that B fertilizer can increase root sugar content by promoting carbohydrate transport (Gezgin et al., 2001). Additionally, boron application reduces sugar loss by lowering beet root amino N, K, and Na contents, ultimately increasing the desired sugar content (Makhlouf et al., 2020). The current investigation, involving slow-release B fertilizer application in the black soil region, showed that the application of slow-release B fertilizers facilitated a synergistic improvement in both optimal biological yield and white sugar yield. The main reason is that granular B fertilizer is more effective in improving root properties in both soil types. This was evidenced by the fact that B application lowered Na and amino N contents while increasing Pol and white sugar contents. Additionally, white sugar content was found to be strongly negatively correlated with Na and amino N contents. These results indicate that slow-release B fertilizer can improve quality characteristics, such as sugar content, amino N, and Na in beet root, by ensuring an effective supply of B during the late growth period. This, in turn, reduces sugar loss during processing and ultimately increases sugar yield (**Figure 6**). This research provides guidance for the application of new types of B fertilizer and the improvement of sugar beet white sugar yield.

## 5 Conclusions

Slow-release B fertilizer significantly increased the boron transfer coefficient of two sugar beet varieties during the later growing stage in the black soil zone of Northeast China. This enhancement in the boron transfer coefficient improved photosynthetic performance and promoted the transport of dry matter to the roots, resulting in higher root yield. Boron application also increased root B accumulation and reduced ash content, thereby improving root quality and increasing white sugar yield. Our findings provided a scientific foundation for developing novel and effective B-based fertilizers for sugar beet in Northeast China. The outcomes of this research offer a general and theoretical framework for improving the yield and quality of root crops.

## Data Availability

The original contributions presented in the study are included in the article/**Supplementary Material**. Further inquiries can be directed to the corresponding authors.
