# Supplementary material for: Slow-release boron fertilizer improves yield and nutritional profile of Beta vulgaris L. grown in Northeast China by increasing boron supply capacity
Source: Front Plant Sci. 2024 Dec 16;15:1441226. doi: 10.3389/fpls.2024.1441226 (PMC11683845; doi:10.3389/fpls.2024.1441226)
Supplement: Supplementary file 1 [file Table1.docx]

| **TABLE S1** The soil chemical characteristics of the two sites in China. | | | | |
| --- | --- | --- | --- | --- |
| Soil properties | Hulan  （BS） | | Baoshan  （AS） | |
|  | 2019 | 2020 | 2019 | 2020 |
| pH | 6.93 | 6.92 | 6.58 | 6.73 |
| Organic matter (g kg^-1^) | 5.32 | 42.40 | 30.20 | 28.35 |
| Total N (g kg^-1^) | 3.52 | 3.32 | 2.90 | 2.75 |
| Total P (g kg^-1^) | 2.10 | 2.03 | 1.09 | 1.13 |
| Total K (g kg^-1^) | 25.50 | 23.40 | 21.60 | 20.50 |
| Alkaline hydrolyzable N (mg kg^-1^) | 116.08 | 111.88 | 168.47 | 153.07 |
| Available P (mg kg^-1^) | 61.20 | 66.41 | 40.38 | 33.02 |
| Available K (mg kg^-1^) | 163.71 | 132.05 | 182.17 | 209.87 |
| Available B (mg kg^-1^) | 0.33 | 0.29 | 0.29 | 0.45 |
| Note: BS, and AS represent black soil and albic soil, respectively. | | | | |
