# Supplementary material for: Slow-release boron fertilizer improves yield and nutritional profile of Beta vulgaris L. grown in Northeast China by increasing boron supply capacity
Source: Front Plant Sci. 2024 Dec 16;15:1441226. doi: 10.3389/fpls.2024.1441226 (PMC11683845; doi:10.3389/fpls.2024.1441226)
Supplement: Supplementary file 2 [file Table2.docx]

| **TABLE S2 Calculation formula** |
| --- |
| Leaf area (cm^2^ plant^-1^) = single leaf area (cm^2^) × leaf dry weight (g plant^-1^)/ single leaf dry weight (g) |
| Leaf area index (LAI) = Total leaf area (cm^2^ plant^-1^) / land area covered by plant leaves (cm^2^ plant^-1^) |
| Standard molasses loss (%) = 0.12 (K + Na) + 0.24 × amino N + 1.08, where K, Na and amino N were described in mmol (100g)^-1^ |
| White sugar content (%) = sucrose (%) - standard molasses loss (%) |
| White sugar yield (t ha^-1^) = root yield (t ha^-1^) × white sugar content (%) |
